# Epidemiologists and causation in an intricate world

**DOI:** 10.1186/1742-7622-2-3

**Published:** 2005-05-24

**Authors:** Mauricio L Barreto

**Affiliations:** 1 Instituo de Saúde Coletiva Universidade Federal da Bahia Salvador, Bahia, Brazil

## 

The delivery of treatments for diseases has always been the central mission of medicine as the delivery of preventative or control measures has always been the mission of public health. This capacity to deliver interventions has been present over the history of both disciplines, independent of the degree of knowledge of causes of diseases or effectiveness of treatment or preventative measures.

The development and reaffirmation of epidemiology as a scientific discipline has been closely associated with its ability to search for causes of health-related events. Causal reasoning was always part of human thinking and of philosophical concerns. However, the first to transform philosophical causal concerns in an organized logical system from which causal relationships could be inferred was the philosopher J. Stuart Mill in the mid-19^th ^century. He formulated the so called Mill's Canons [1]. At the same time, bacteriologists excited with their newly-emerging discipline were looking for more specific systems to help them to check etiological associations between infectious agents and specific diseases, and to attend this need the Henlé-Kock postulates were formulated. After that, it would take nearly a century for new causal formulations related to health-related events to be developed. The rise of the chronic diseases as the main component of the morbidity and the mortality in rich countries and the consequent changes that this had upon the causal thinking in epidemiology (until then dominated by infectious disease) was the stimulus for the development of the widely known Hill criteria of causality [2]. In order to address problems raised by newly discovered infectious agents that did not behave according to the Henlé-Kock postulates and in an effort to unify the causal verification of infectious and non-infectious health problems, Evans [3] presented a new set of postulates.

To make the lives of epidemiologists difficult, the causes of health-related events are multiple and very heterogeneous – varying from a micro-infectious agent to a macro-social factor. Ideas of multicausality have long been part of epidemiological thinking, and multi-causal models have been built-up. However, the common way epidemiology search for causes continue to be throughout the test of one-by-one potential causes, even if it is part of a multi-causal complex where grouping all the others causes are grouped under the general label of confounders. It is not by chance that the classical study designs used in epidemiology are well suited for this task.

During the 1990s several leading epidemiologists intensified alerts about the insufficiency and limits of such strategies used in epidemiology [4-8]. While the focus of their criticisms varied from theoretical or paradigmatic questions to more applied ones, together they raised serious concerns about the role of epidemiology to fulfill its mission of producing sound knowledge and informing effective actions compatible with present and future population health needs. As a consequence, this is a special moment in which the clarification of aspects related with causation could help epidemiology to overcome its epistemological difficulties and keep on track with its mission, including the study of the determinants of health-related states or events.

It is worth noting that this is not a crisis restricted to epidemiology; it also affects different disciplines involved in the understanding of human and social events, and the roots of their causes. We are living in a moment when crucial philosophical and scientific questions are being debated. More than ever words like chaos, complexity, dynamic models, etc. have been present in the philosophical, scientific and lay literatures. In this context, the causality debate has been intensified, but now in connection with the complex ways that human and social events are now perceived. Causality is understood as a relational phenomenon, which has theoretical, but also practical implications. A cause can be the presence or the absence of an action depending of the position of the observer. A cause can increase or decrease the occurrence of a health-related event – causation and prevention are different faces of the same coin. A cause is always the outcome of other causes. But, as far prevention is concerned, when suppression or activation of a specific cause is feasible and generates the desired prevention, there is no immediate need to understand its own causes. A cause is an analyzable factor but some times it is also consequence of a deliberate intervention – the quality of environment affects health, but a clean environment can be a natural occurrence in forests or can be the consequence of an intervention directed to decrease pollution in urban places. Similarly, housing is an important factor related to health-events, but it also consequence of programs implemented to change the housing situation of a given population.

Epidemiology has used two different approaches to study causes of the health-related events: the experimental and the observational. Experimental studies (frequently randomized community trials) evaluate a cause by comparing similar groups of individuals exposed and non-exposed to an intervention targeted to suppress or stimulate this cause. This characteristic means that randomized trials are considered by many to be the ultimate standard for definition of a causal association. For some radical minds, the observational approach cannot even be considered in causal discussion! However, for several reasons, including operational and ethical ones, a great part of the causal knowledge accumulated in epidemiology comes from observational studies where very often the comparison groups are not similar or even do not exist. Consider the situation where the vaccine *X *is a "cause". While it is possible to test its effect using observational studies there is a great consensus that this is best done by a randomized trial. However, if the situation of a vaccination program using the same vaccine, a new causal problem is born, and use of a randomized trial is not as feasible as before. And for a great number of causal problems, experimental studies are totally unfeasible. In some special cases, the two approaches could be used complementarily: for instance, the effect of vitamin A deficiency on child morbidity (diarrhea and acute lower respiratory infections) was verified using observational studies and later, the effect of vitamin A supplementation of deficient children on morbidity was tested experimentally.

The questions put forward by Greenland in the paper published here [9] is part of a great effort made by him and others [10-12] to understand the complex nature of causation. In this intricate world, advancements in the understanding of causality is a task that needs to conjugate very wise philosophical (but not metaphysical) and empirical (but not empiricist) perspectives. Epidemiologists have a difficult time as they must see causality as a dynamic and multivariate process, but without losing the opportunities for prevention and without getting lost in the web of causation.

## Competing interests

The author(s) declare that they have no competing interests.
